# The SA-WRKY70-PR-Callose Axis Mediates Plant Defense Against Whitefly Eggs

**DOI:** 10.3390/ijms252212076

**Published:** 2024-11-10

**Authors:** Hong-Da Song, Feng-Bin Zhang, Shun-Xia Ji, Xue-Qian Wang, Jun-Xia Wang, Yu-Xiao Liu, Xiao-Wei Wang, Wen-Hao Han

**Affiliations:** State Key Laboratory of Rice Biology and Breeding, Ministry of Agriculture Key Lab of Molecular Biology of Crop Pathogens and Insects, Zhejiang Key Laboratory of Biology and Ecological Regulation of Crop Pathogens and Insects, Institute of Insect Sciences, Zhejiang University, Hangzhou 310058, China; 19970409@zju.edu.cn (H.-D.S.); zhangfengbin@zju.edu.cn (F.-B.Z.); shunxia-ji@zju.edu.cn (S.-X.J.); xqwang@zju.edu.cn (X.-Q.W.); wangjunxia08@zju.edu.cn (J.-X.W.); yuxiaoliu@zju.edu.cn (Y.-X.L.); xwwang@zju.edu.cn (X.-W.W.)

**Keywords:** plant–insect interaction, insect eggs, *Bemisia tabaci*, phytohormone, callose deposition

## Abstract

The molecular mechanisms of plant responses to phytophagous insect eggs are poorly understood, despite their importance in insect–plant interactions. This study investigates the plant defense mechanisms triggered by the eggs of whitefly *Bemisia tabaci*, a globally significant agricultural pest. A transcriptome comparison of tobacco plants with and without eggs revealed that whitefly eggs may activate the response of defense-related genes, including those involved in the salicylic acid (SA) signaling pathway. SA levels are induced by eggs, resulting in a reduction in egg hatching, which suggests that SA plays a key role in plant resistance to whitefly eggs. Employing *Agrobacterium*-mediated transient expression, virus-induced gene silencing assays, DNA–protein interaction studies, and bioassays, we elucidate the regulatory mechanisms involved. Pathogenesis-related proteins NtPR1-L1 and NtPR5-L2, downstream of the SA pathway, also affect whitefly egg hatching. The SA-regulated transcription factor NtWRKY70a directly binds to the *NtPR1-L1* promoter, enhancing its expression. Moreover, NtPR1-L1 promotes callose deposition, which may impede the eggs’ access to water and nutrients. This study establishes the SA-WRKY70-PR-callose axis as a key mechanism linking plant responses and defenses against whitefly eggs, providing new insights into the molecular interactions between plants and insect eggs.

## 1. Introduction

Phytophagous insects typically deposit their eggs on host plants, marking the initiation of a new herbivore generation and posing a significant threat to the host. Despite their seemingly inert presence, insect eggs have been shown to exert various effects on host plants [1]. Oviposition by a diverse array of insect taxa, including butterflies, moths, sawflies, beetles, bugs, and planthoppers, has been demonstrated to elicit plant defense responses [1,2].

In response, host plants have evolved a range of inducible defenses specifically targeted against insect eggs. These defenses include early immune responses mediated by phytohormones, defense-related genes, and reactive oxygen species (ROS), as well as visible egg-killing mechanisms such as hypersensitive response (HR), necrosis, egg-crushing, and ovicidal substances [3,4,5,6,7]. The phytohormones jasmonic acid (JA) and salicylic acid (SA) have been implicated in the plant responses to insect eggs. For instance, oviposition by the tomato fruitworm moth, *Helicoverpa zea*, triggers a JA burst in tomato plants, which is associated with a wound response [8]. Similarly, SA accumulates at the site of egg deposition by the large cabbage white butterfly, *Pieris brassicae*, in *Arabidopsis* leaves [9]. Egg extracts from certain species are capable of inducing SA levels in plants; for example, egg extracts from *P. brassicae* can affect *Arabidopsis* and *Brassica nigra,* while extracts from the sawfly *Diprion pini* and the beetle *Xanthogaleruca luteola* influence *Arabidopsis* [7,10]. Moreover, transcriptional analyses have indicated that JA- and SA-related genes are involved in host responses to eggs from various insects [11,12,13]. Notably, while significant attention has been given to the roles of JA and SA, research on phytohormone-induced resistance specifically to eggs remains limited.

Phytophagous insects exhibit diverse oviposition behaviors on host plants [1,14]. For instance, adult female lepidopterans lay eggs without feeding or causing noticeable damage to the host. In contrast, female coleopterans may cause significant harm during egg laying. Some hemipteran insects, on the other hand, lay eggs while simultaneously feeding on phloem sap using their specialized stylet, making it difficult to differentiate between feeding and oviposition behaviors. Additionally, insect eggs adhere to the host plants in various ways: some adhere to the surface, others are laid within the wounds caused by oviposition, and some connect directly via an egg pedicel inserted into the tissue [1,15]. Thus far, most studies on host-egg interactions have focused on insects with tissue-chewing larvae or adults, such as moths, butterflies, beetles, and sawflies, while investigations into how host plants respond to eggs from phloem-feeding insects, particularly at the transcriptional and phytohormone levels, remain limited [1,2].

**Figure 1:**
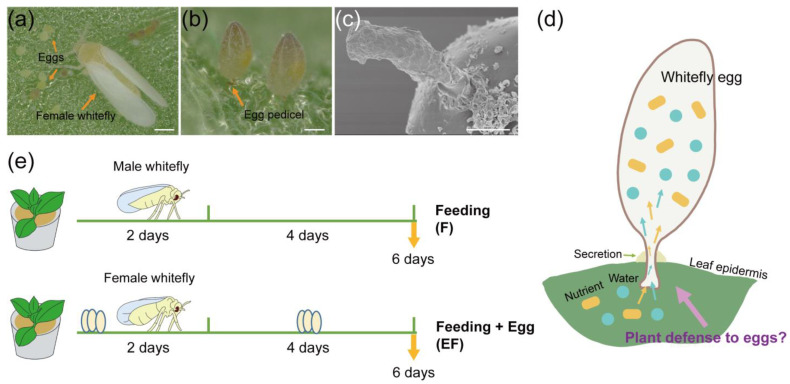
The whitefly egg–plant interaction and experimental design. (**a**) Whitefly feeding and laying eggs on the abaxial leaf surface of tobacco plants. Orange arrows indicate a female whitefly and eggs respectively. Scale bar, 200 μm. (**b**) Whitefly egg anchored in tobacco leaf by an egg stalk (orange arrow), called a pedicel. Scale bar, 50 μm. (**c**) A whitefly was allowed to lay eggs on an extended parafilm, then the eggs were removed intact from the parafilm by conductive tape. The scanning electron micrograph shows the characteristics of whitefly egg pedicel. Scale bar, 10 μm). (**d**) Interaction between whitefly eggs and tobacco leaves. Blue circles represent water and orange rounded rectangles represent nutrients. (**e**) Study experimental design. Male and female whiteflies were respectively collected and released to tobacco plants to feed (male) and to feed and oviposit (female) for 2 d. Then, adults were removed and tobacco leaves were sampled on day 6.

The whitefly *Bemisia tabaci* (Gennadius), a phloem-feeding insect, is known as a species complex likely containing > 40 cryptic species, some of which are significant crop pests worldwide [16,17,18,19]. Adult female whiteflies can produce hundreds of eggs during their 2–3 week lifespan [20,21], posing a significant challenge to host plants. They oviposit and feed simultaneously, inserting eggs into the leaf epidermal cells with a glue-like secretion from their collateral glands to secure eggs in place (Figure 1a–c) [22]. During egg development, water and potential nutrients from the leaf are absorbed through the egg pedicel [23,24], suggesting a potential avenue for regulating plant defense (Figure 1d). However, the processes by which whitefly eggs induce plant responses and regulate host defenses remain unclear.

In this study, we investigated the transcriptional and phytohormone responses of tobacco plants to whitefly eggs infestation. We demonstrated that SA accumulation is induced by whitefly eggs and reduces egg hatching. Additionally, we identified two *pathogenesis-related* (*PR*) genes downstream of the SA signaling pathway that participate in egg resistance. We also characterized an SA-regulated transcription factor, NtWRKY70a, which directly binds to the promoter of *NtPR1-L1*, activating its expression. Furthermore, we provided evidence for the potential mechanism by which NtPR1-L1 affects eggs through enhancing callose accumulation. These findings suggest that the SA-WRKY70-PR-callose axis plays a crucial role in linking host plant responses and defenses against insect eggs.

## 2. Results

### 2.1. Whitefly Eggs Induce Transcriptional Responses in Tobacco

**Figure 2:**
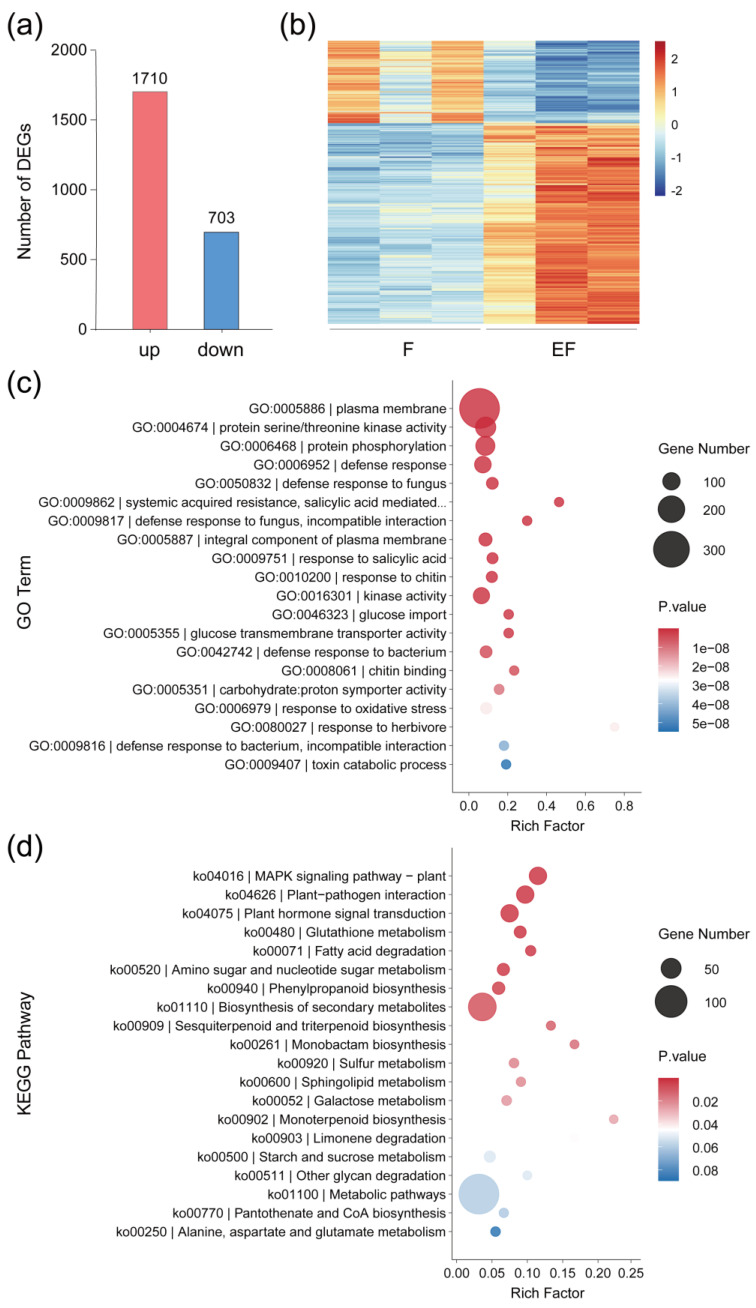
Tobacco transcriptome responses to whitefly eggs and feeding. (**a**) Histogram showing up- and downregulated number of DEGs between eggs + feeding samples (EF) vs. feeding samples (F). (**b**) Heatmap showing the expression patterns of DEGs between F and EF. Each treatment had three replicates. The red to blue colors represent high to low expression levels based on the Log10-transformed FPKM values. (**c**,**d**) Enrichment plot showing top 20 *p*-value GO terms (**c**) and KEGG pathways (**d**) of upregulated DEGs between F and EF.

Female whiteflies feed and oviposit simultaneously, making it difficult to differentiate between egg-laying and feeding. To minimize the impact of feeding effects and identify potential genes induced by eggs, we used tobacco as the host plant and designed an experiment including three treatments: feeding (F), eggs + feeding (EF), and untreated control (C) (Figure 1e). Compared to the F treatment, the EF treatment exhibited 1710 upregulated and 703 downregulated genes (Figure 2a). The expression patterns of these differentially expressed genes (DEGs) are visualized in a heatmap (Figure 2b), with detailed information (e.g., gene_id and FPKM) provided in Table S1. The RNA-seq results of the F vs. C comparison showed fewer DEGs, with 218 upregulated and 333 downregulated genes (Figure S1). These results indicate that the transcriptional response in tobacco plants largely recovered from feeding, suggesting that the upregulated DEGs obtained from the EF vs. F comparison were largely induced by the oviposition behavior and the presence of eggs.

The upregulated DEGs from the EF vs. F comparison were categorized into various Gene Ontology (GO) terms and Kyoto Encyclopedia of Genes and Genomes (KEGG) pathways. The top GO terms analysis in the biological process revealed that the DEGs were primarily associated with various stress responses (e.g., defense responses to bacteria and fungi), response to SA, and systemic acquired resistance (Figure 2c). The top KEGG pathways analysis indicated that the most significant egg-inducible DEGs were related to biosynthesis (e.g., secondary metabolite and phenylpropanoid biosynthesis) and signaling pathways (e.g., MAPK signaling, plant hormone signal transduction) (Figure 2d). These results suggest that whitefly eggs may trigger defense-related pathways and gene responses in plants.

### 2.2. Whitefly Eggs Induce Salicylic Acid (SA) Accumulation but Not Jasmonic Acid (JA) in Tobacco

**Figure 3:**
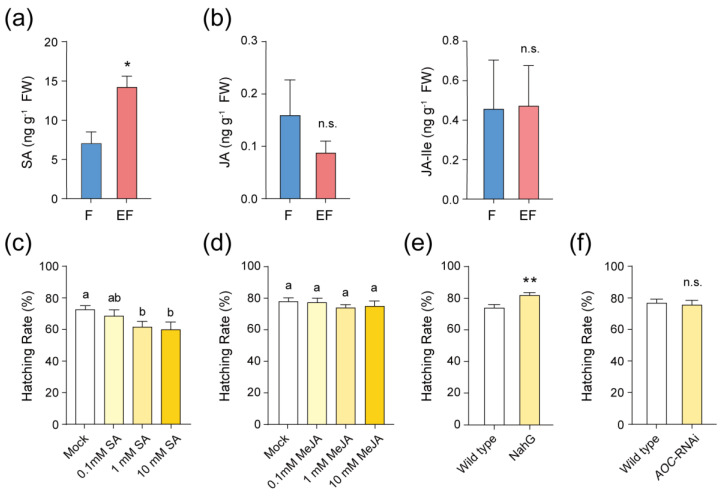
Egg-inducible SA accumulation reduces the hatching of eggs. (**a**,**b**) Concentrations of SA (**a**), JA, and JA-Ile (**b**) in feeding samples (F) and eggs + feeding samples (EF). *n =* 6. Values are mean ± SEM. n.s., not significant; *, *p* < 0.05 (Student’s *t*-test). (**c**,**d**) Egg-hatching rates on tobacco plants upon exogenous application of 0.1 mM, 1 mM, 10 mM SA (**c**), and MeJA (**d**). *n =* 15. Values are mean ± SEM. Bars with different lowercase letters indicate significant differences between treatments at *p* < 0.05 (one-way ANOVA, LSD test). (**e**,**f**) Egg-hatching rates on NahG (**e**) and *AOC*-RNAi (**f**) tobacco. *n =* 30. Values are mean ± SEM. n.s., not significant; **, *p* < 0.01 (Student’s *t*-test).

Our analysis revealed significant changes in the expression patterns of genes associated with the SA signaling pathway, while the JA-related genes remained largely unchanged (Table S2). To determine whether whitefly eggs could induce SA or JA accumulation, we measured the concentrations of SA, JA, and JA-Isoleucine (JA-Ile) in the EF and F treatments, employing the same experimental design as in the RNA-seq (Figure 1e). The results showed that SA accumulation was significantly higher in the EF samples than in the F sample (Figure 3a), while JA and JA-Ile levels remained low and relatively unchanged (Figure 3b). These findings suggest that whitefly eggs induce SA accumulation in tobacco without significantly affecting JA levels.

### 2.3. SA Accumulation Affects Whitefly Egg Hatching

We hypothesized that the SA accumulation induced by whitefly eggs plays a role in plant defense against these eggs. To test this hypothesis, SA and methyl jasmonate (MeJA) were applied to wild-type tobacco plants, and the hatching rates of whitefly eggs were evaluated. The data indicated a significant reduction in egg hatching when 1 mM and 10 mM SA were applied (Figure 3c). In contrast, MeJA application at the same concentrations had no significant effect on egg hatching (Figure 3d). To further explore the role of SA in tobacco’s defense against whitefly eggs, NahG tobacco plants, which are impaired in intrinsic SA accumulation, were introduced for experimentation. In these plants, the hatching rate of whitefly eggs was higher compared to wild-type plants (Figure 3e). However, in *AOC*-RNAi tobacco plants, which are deficient in JA accumulation, the egg-hatching rate remained comparable to that of wild-type plants (Figure 3f). These results demonstrate that SA accumulation, rather than JA, influences the hatching of whitefly eggs.

### 2.4. SA-Responsive NtPRs Are Involved in Response and Defense to Whitefly Eggs

**Figure 4:**
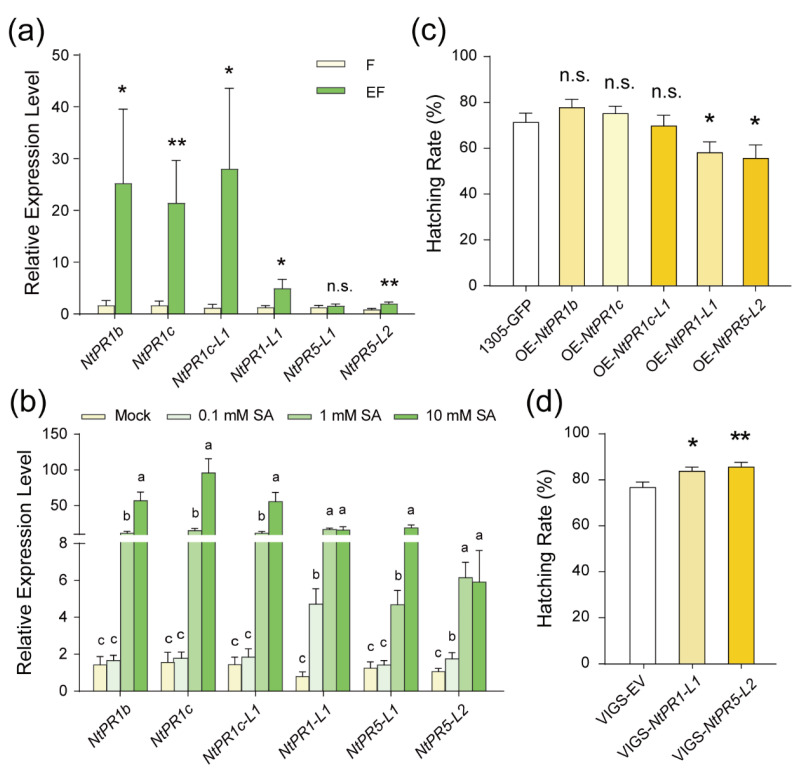
SA-responsive *NtPRs* are involved in response and defense to whitefly eggs. (**a**,**b**) The relative transcription levels of six *NtPRs* in feeding samples (F) and eggs + feeding samples (EF) of tobacco plants (a, *n =* 8, *, *p* < 0.05, Student’s *t*-test), and in plants following application of 0.1 mM, 1 mM, 10 mM SA, and mock (b, *n =* 6, *p* < 0.05, one-way ANOVA, LSD test). Values are mean ± SEM of two technical replicates. n.s., not significant. (**c**,**d**) Egg-hatching rates on five *NtPRs* over-expression (OE) (**c**) and two VIGS-*NtPRs* (**d**) tobacco plants. Values are mean ± SEM, *n =* 15. n.s., not significant; *, *p* < 0.05; **, *p* < 0.01 (Student’s *t*-test).

SA is a key signaling molecule regulating various plant defense mechanisms [25,26]. To understand how SA enhances tobacco defense against eggs, we focused on genes that respond to both whitefly eggs and SA accumulation. *PR* genes, particularly *PR1* and *PR5*, are recognized as downstream responsive genes of SA [26], so we examined the expression patterns of *NtPR1* and *NtPR5*. Using RNA-seq data, we identified four *PR1* family genes and two thaumatin-like genes from the *PR5* family to be upregulated in the EF samples compared to the F samples (Figure S2). Subsequent RT-qPCR analysis confirmed that six of these *NtPR* genes were significantly upregulated (Figure 4a). Further investigation revealed that all *NtPRs* were upregulated by 1 mM or 10 mM SA (Figure 4b). Thus, five *NtPRs* (*NtPR1b*, *NtPR1c*, *NtPR1c-L1*, *NtPR1-L1*, and *NtPR5-L2*) were likely induced by both whitefly eggs and SA.

To evaluate the role of these *NtPR* genes in plant resistance to whitefly eggs, we individually overexpressed these five genes in tobacco using *Agrobacterium*-mediated transformation. Bioassay results revealed that two of them, *NtPR1-L1* and *NtPR5-L2*, significantly influenced egg hatching when overexpressed (Figure 4c). To further assess their role, we used virus-mediated gene silencing (VIGS) to knock down the expression of *NtPR1-L1* and *NtPR5-L2*. RT-qPCR analysis confirmed the reduced expression of these genes in the silenced tobacco plants (Figure S3). The egg-hatching rate was significantly higher on the VIGS-treated plants compared to the control (Figure 4d). These findings demonstrate that the SA-responsive genes *NtPR1-L1* and *NtPR5-L2* play a crucial role in SA-mediated defense against whitefly eggs.

### 2.5. SA-Responsive NtWRKY70a Participates in Plant Defense Against Whitefly Eggs

**Figure 5:**
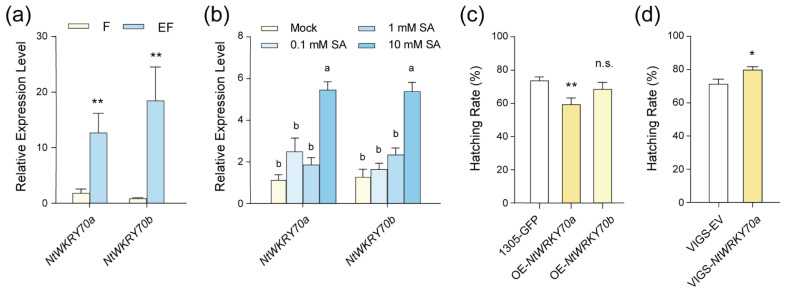
SA-responsive *NtWRKY70a* participates in plant defense against whitefly eggs. (**a**,**b**) The relative transcription levels of two *NtWRKY70s* in feeding samples (F) and eggs + feeding samples (EF) of tobacco (a, *n =* 8, *, *p* < 0.05, Student’s *t*-test), and in plants following application of 0.1 mM, 1 mM, 10 mM SA, and mock (b, *n =* 6, *p* < 0.05, one-way ANOVA, LSD test). Values are mean ± SEM of two technical replicates. (**c**,**d**) Egg-hatching rate on two *NtWRKY70s* over-expression (OE) (**c**) and VIGS-*NtWRKY70* (**d**) tobacco plants. Values are mean ± SEM, *n =* 15. n.s., not significant; *, *p* < 0.05; **, *p* < 0.01 (Student’s *t*-test).

To further explore how SA regulates the expression of *NtPR1-L1* and *NtPR5-L2* in the plant’s response to whitefly eggs, we focused on transcription factors (TFs) that may be involved in this process. Previous studies indicated that the TF *WRKY70*, downstream of the SA signaling pathway, can regulate *PR1* and *PR5* gene activation in *Arabidopsis* [27]. Based on this, we screened for *NtWRKY70* genes involved in the response and defense against whitefly eggs. RNA-seq analysis revealed that two *NtWRKY70*-*like* genes, designated *NtWRKY70a* and *NtWRKY70b*, were upregulated in EF samples compared to F (Figure S4). Subsequent RT-qPCR tests confirmed their significant induction by eggs (Figure 5a). Additionally, SA application assays demonstrated that both *NtWRKY70a* and *NtWRKY70b* were regulated by SA (Figure 5b). These results suggest that *NtWRKY70a* and *NtWRKY70b* are induced by both whitefly eggs and SA.

To assess whether *NtWRKY70* genes are involved in resistance to whitefly eggs, we individually overexpressed both *NtWRKY7a* and *NtWRKY70b* in tobacco plants. Bioassay results indicated that *NtWRKY70a* influenced egg hatching when overexpressed (Figure 5c). To further validate the role of *NtWRKY70a*’s role in egg resistance, we used VIGS to suppress its expression. RT-qPCR confirmed that the gene expression in *NtWRKY70*-silenced tobacco plants was significantly lower than in control (Figure S5), and the egg hatching was significantly higher on these silenced plants (Figure 5d). These findings identify *NtWRKY70a*, a SA-regulated transcription factor, as a crucial component of tobacco’s defense mechanism against whitefly eggs.

### 2.6. NtWRKY70a Activates NtPR1-L1 Transcription by Binding to Its Promoter

**Figure 6:**
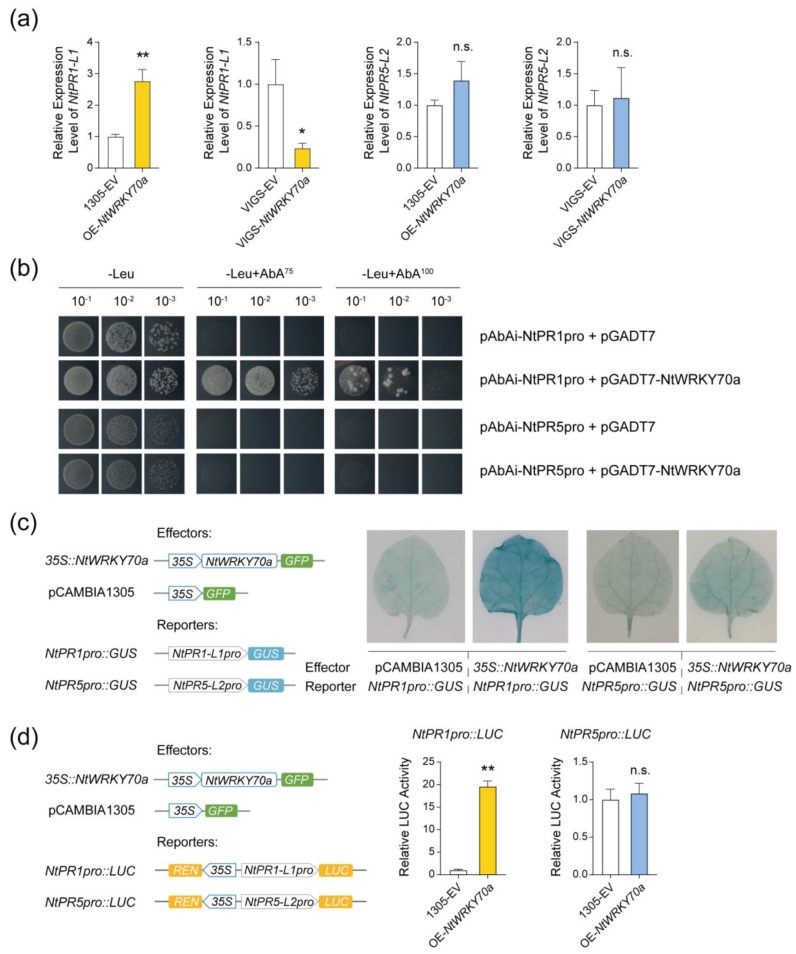
NtWRKY70a activates the transcription of *NtPR1-L1* by binding to its promoter. (**a**) Relative expression levels of *NtPR1-L1* and *NtPR5-L2* in the *NtWRKY70a* over-expression (OE) and VIGS-*NtWRKY70a* tobacco plants. Values are mean ± SEM of two technical replicates, *n =* 8–14. n.s., not significant; *, *p* < 0.05; **, *p* < 0.01 (Student’s *t*-test). (**b**) Yeast one-hybrid assay of the interaction between *NtPRs* promoter and *NtWRKY70a*. Gold yeast cells co-transformed with pAbAi-NtPRpro and pGADT7-NtWRKY70a plasmids were cultured on SD/-Leu medium with or without 75 ng ml^−1^ or 100 ng ml^−1^ AbA for 3 d. The empty vector pGADT7 was used as a negative control. (**c**) Analysis of the interaction between *NtPRs* promoter and NtWRKY70a in tobacco leaves using GUS staining assay. Diagrams on the left show the reporter and effector vectors. *35S*, *CaMV 35S* promoter; *GFP*, *green fluorescent protein*; *GUS*, *β-glucuronidase*. Representative photographs from five to six replicates are shown. (**d**) Analysis of the interaction between *NtPRs* promoter and NtWRKY70a in tobacco leaves using a dual-luciferase reporter assay. Diagrams on the left show the reporter and effector vectors. *REN*, *Renilla luciferase*; *LUC*, *firefly luciferase*. The activity of REN was used as an internal control. Values are mean ± SEM, *n =* 3–6. n.s., not significant; **, *p* < 0.01 (Student’s *t*-test).

We hypothesized that NtWRKY70a affects egg hatching on tobacco by regulating *NtPR* gene expression. To investigate the subcellular localization of *NtWRKY70a*, we overexpressed a *NtWRKY70a*-GFP fusion in the leaves of *Nicotiana benthamiana*. The results showed that the NtWRKY70a protein was localized in the nucleus (Figure S6). RT-qPCR analysis results showed that the transcription level of *NtPR1-L1* was enhanced in *NtWRKY70a*-overexpressing tobacco and suppressed in *NtWRKY70a*-silenced tobacco, while the transcription level of *NtPR5-L2* remained comparable among treatments (Figure 6a). Thus, we hypothesized that *NtWRKY70a* activates the transcription of *NtPR1-L1* but not *NtPR5-L2*.

To test this hypothesis, we conducted a yeast one-hybrid (Y1H) assay. The yeast strain carrying both pAbAi-NtPR1pro and pGADT-NtWRKY70a constructs grew on the SD/-Leu medium with or without AbA at the concentration of 75 ng mL^−1^ or 100 ng mL^−1^. However, the yeast strain co-transformed with pAbAi-NtPR5pro and pGADT-NtWRKY70a constructs failed to grow in the presence of ABA. These results indicate that NtWRKY70a specifically interacts with the *NtPR1-L1* promoter, but not with the *NtPR5-L2* promoter in yeast cells (Figure 6b). Further validation was obtained through GUS staining and dual-luciferase reporter assays. When *35S::NtWRKY70a* (effector) was co-expressed with *NtPR1pro::GUS* (reporter) in tobacco leaves, GUS expression was enhanced. In contrast, co-expression with the *NtPR5-L2* promoter did not result in any significant change in activity confirming that *NtWRKY70a* activates the *NtPR1-L1* promoter but not *NtPR5-L2* (Figure 6c). In dual-luciferase reporter assay, the LUC/REN ratio was significantly upregulated when *NtPR1pro::LUC* was co-expressed with *35S::NtWRKY70a* (Figure 6d), further confirming the direct activation of *NtPR1-L1* by NtWRKY70a. Collectively, these results demonstrate that NtWRKY70a directly activates the transcription of *NtPR1-L1* by binding to its promoter.

### 2.7. NtPR1-L1 Contributes to Egg Resistance by Inducing Callose Deposition

**Figure 7:**
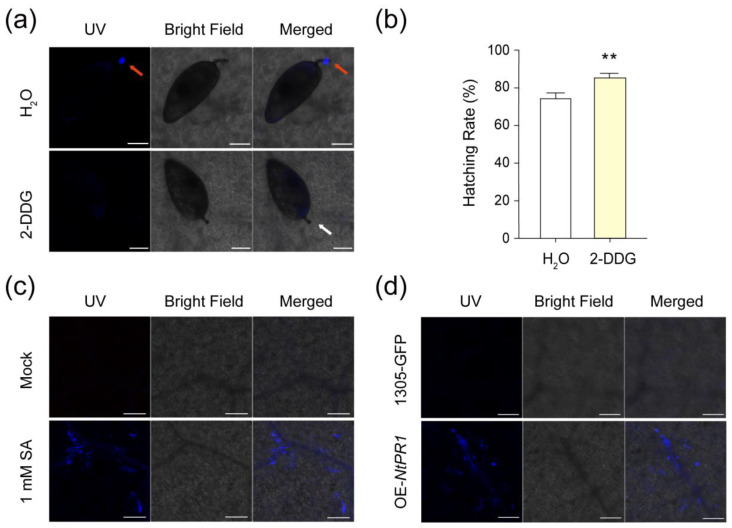
*NtPR1-L1* contributes to egg resistance by inducing callose deposition. (**a**) Callose staining assay of the egg-induced callose deposition in tobacco leaves treated with 2-deoxy-D-glucose (2-DDG) and H_2_O, respectively. Red arrows indicate the callose deposition around the pedicel, and white arrow indicates a pedicel without callose deposition. Scale bars, 50 μm. (**b**) Egg-hatching rate on 2-DDG- and H_2_O-treated tobacco plants. Values are mean ± SEM, *n =* 15. **, *p* < 0.01 (Student’s *t*-test). (**c**) Callose staining assay of tobacco plants treated with 1 mM SA for 5 d. Scale bars, 50 μm. Representative results from five replicates are shown. (**d**) Callose staining assay of *NtPR1* over-expressing (OE) tobacco plants. 1305-GFP was used as a control. Scale bars, 50 μm. Representative results from five replicates are shown.

Callose, a polysaccharide that accumulates in response to various biotic and abiotic stresses, plays a key role in plant defense mechanisms [28,29]. Previous studies have demonstrated that callose deposition is induced in whitefly-infested tissues, acting as a barrier that prevents whiteflies from continuously ingesting phloem sap [30,31]. In this study, we observed that whitefly eggs induced callose deposition around the egg pedicel (Figure 7a). The results showed that callose deposition was inhibited when treated with the callose synthesis inhibitor 2-deoxy-D-glucose (2-DDG). Notably, compared to the H_2_O-injected plants, the egg-hatching rate increased significantly in 2-DGG-injected plants, indicating that callose reduces egg hatching (Figure 7b).

To investigate whether SA induces callose deposition, we sampled tobacco treated with 1 mM SA and stained with aniline blue. The results demonstrated that SA treatment significantly induced callose deposition in tobacco leaves (Figure 7c). Callose staining assays also showed that overexpressing of *NtPR1-L1* increased callose deposition (Figure 7d). Callose deposition is primarily regulated by *callose synthase* (*CalS*) gene family. In *Arabidopsis*, several *CalS* genes, including *CalS1*, *CalS3*, *CalS7*, *CalS8,* and *CalS12*, have been implicated in plant defense responses [29,32,33]. Compared to the control, the transcription levels of all *NtCals* genes remained unchanged, while a *β-1,3-glucanase* (*BG*) gene responsible for callose degradation was downregulated in *NtPR1-L1*-overexpressing plants (Figure S7). Additionally, *NtWRKY70a* was also found to promote callose accumulation (Figure S8). Together, these results suggest that SA, *NtWRKY70a*, and *NtPR1-L1* may enhance plant resistance against whitefly eggs by positively regulating callose deposition.

## 3. Discussion

In this study, we investigated the transcriptional response of tobacco to whitefly eggs, designing RNA-seq sampling methods based on whitefly feeding and oviposition characteristics (Figure 1e). By comparing gene expression between the EF and F treatments, we identified DEGs that were likely induced by whitefly eggs. GO and KEGG enrichment analyses revealed the upregulation of genes associated with plant stress responses, defense mechanisms, secondary metabolite synthesis, signal transduction, and plant–pathogen interaction pathways (Figure 2). Our findings suggest that plant responses to insect eggs involve multiple stress-related pathways, encompassing both biotic and abiotic stress responses. Previous investigations on plant–insect egg interactions have shown that insect eggs can activate defense-related genes across different host plants, indicating common transcriptional responses [11,12,13,34,35,36,37,38,39]. Collectively, these studies, along with our findings, suggest a degree of conservation in the plant defense mechanisms against various insect eggs, despite differences in oviposition patterns.

**Figure 8:**
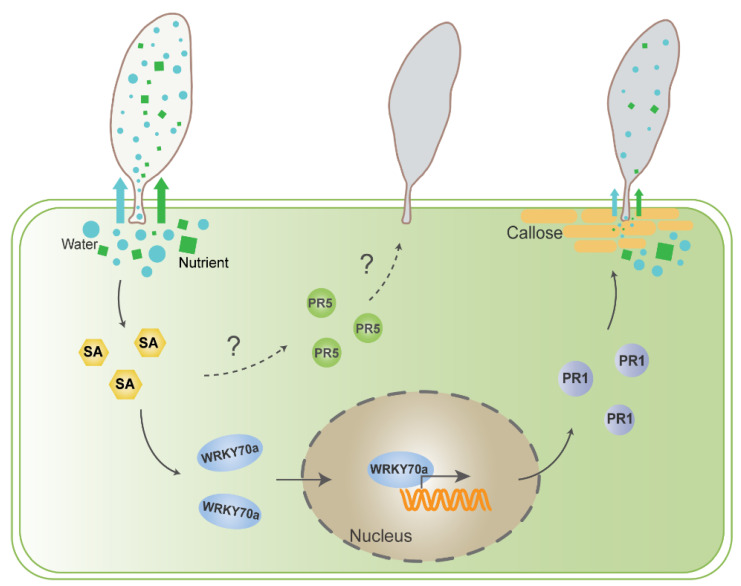
A model depicting the functional relationships among SA, NtWRKY70a, and NtPRs, and their roles in the plant–whitefly egg interaction. Whiteflies oviposit on plants by inserting and securing eggs into the epidermal tissue. Then, eggs develop by absorbing water and possibly nutrients through the pedicel until hatching. During this process, SA accumulation in the plant is induced by eggs, then activates the downstream *NtWRKY70a*. As a transcription factor, NtWRKY70a then directly binds to the promoter of *NtPR1-L1* and activates its transcription. *NtPR1-L1* may induce callose deposition, which interferes with the egg’s absorption of substances from the host and affects egg hatching. Moreover, egg-induced SA accumulation may upregulate the transcription of egg resistance gene *NtPR5-L2*, and this regulatory mechanism remains to be determined. PR1 represents NtPR1-L1, PR5 represents NtPR5-L2. Blue circles represent water, green squares represent nutrient, orange rounded rectangles represent callose.

Previous studies have demonstrated that SA and JA play significant roles in plant responses to insect eggs. For example, oviposition by the beetle *X. luteola* significantly increases the expression of JA synthesis genes in elm trees [11], while *P. brassicae* oviposition induces upregulation of JA and SA-related genes in *Arabidopsis* [37]. However, much of this research has focused on the eggs of chewing insect, with limited attention to the eggs of phloem-feeding insects, aside from the brown planthopper, *Nilaparvata lugens*, whose egg-associated secretions induce JA and JA-Ile but not SA in rice [40]. In contrast, our study found that whitefly eggs induce SA accumulation and upregulation of SA-related genes in tobacco plants (Figure 3a, Table S2, Figure 8), while levels of JA, JA-Ile, and expression of JA-related genes remained largely unchanged (Figure 3b, Table S2).

Interestingly, similar results have been observed with butterfly eggs. Lortzing et al. [41] reported that *P. brassicae* eggs also induce SA accumulation in *Arabidopsis* without affecting JA and JA-Ile levels. JA-related gene *lipoxygenase* (*LOX2*) was measured in *B. nigra* too, and was not or only slightly upregulated [42]. Previous research also highlighted the necessity of SA in mediating resistance against subsequent caterpillars or pathogens in *Arabidopsis* following treatment with *P. brassicae* eggs [41,43].

In our study, we employed exogenous phytohormone applications and phytohormone-deficient mutants to further investigate the role of phytohormones in plant resistance to insect eggs. Our data show that the SA signaling pathway reduces whitefly egg hatching, whereas the JA signaling pathway does not affect the hatching rate (Figure 3c–f). Thus, we propose that SA plays a crucial role in responding to and conferring resistance against whitefly eggs, thereby broadening our understanding of the phytohormone’s roles in plant defenses against insect eggs.

PR proteins play critical roles in plant defense response against pathogens. However, their roles in defense against insect eggs remain inadequately understood. Notably, *PR1* and *PR5* are downstream responsive genes of SA [44,45]. Several studies indicate that *PR1* and *PR5* respond to eggs of diverse insect species across various host plants. For instance, *P. brassicae* oviposition induces upregulation of *PR5* in *Arabidopsis* [37] and *PR1* in *B. nigra* [42]; elm *PR1* is induced by elm leaf beetle egg treatment [11]; oviposition of bruchid beetles (*Callosobruchus* spp.) induces upregulation of *PR1* and *PR5* in black gram pods [38]. Similarly, oviposition by the sawfly *D. pini* leads to increased expression of *PR1* and *PR5* in pine [46]. Additionally, the egg secretions of *D. pini*, *P. brassicae*, and *X. luteola* have been shown to upregulate the expression of *PR1* and *PR5* [7].

In this study, we observed that eggs from the phloem-feeding whitefly induce the expression of *NtPR1-like* and *NtPR5-like* genes in tobacco (Figure 4a). This finding suggests that the response of *PR* genes represents a common transcription change in the interaction between various plants and insect eggs. Furthermore, our data show that overexpression of *NtPR1-L1* and *NtPR5-L2* in tobacco reduces whitefly egg hatching, while silencing these genes promotes egg hatching (Figure 4c,d). These findings highlight the resistance conferred by *PR* genes against insect eggs.

The WRKY TF family is widely involved in various defense response pathways in plants and serves as a crucial regulator in defense against insects [47,48,49]. *WRKY* genes are also involved in plant responses to insect eggs; for instance, 17 *WRKYs* in *Arabidopsis* are upregulated in response to oviposition by *P. brassicae* [37]. In our study, we observed that *NtWRKY70* genes are significantly upregulated in response to whitefly eggs (Figure 5a, Figure S4). Through gene overexpression and silencing, we determined their roles in defending against whitefly eggs (Figure 5c,d), thereby expanding our understanding of WRKY TFs in plant–insect interactions. Notably, we observed that *WRKY70a* is regulated by SA and activates downstream *PR1-L1* (Figure 5b, Figure 6 and Figure 8). This parallels the defense mechanisms employed by plants against pathogens, where *WRKY70* can be induced by SA and pathogens infection, activating downstream *PR* genes [50,51]. Furthermore, through molecular assays, we demonstrated that *NtWRKY70a* directly binds to the *NtPR1* promoter to activate its expression (Figure 6b–d). Thus, it is evident that the regulatory mechanisms governing plant defense against whitefly eggs and pathogens share significant similarities.

Callose, a β-1,3-glucan polymer prevalent in higher plants, plays an active role in plant defense responses [52]. When triggered by pathogens, callose forms a protective barrier at the infection site, preventing further invasion [29,53]. Callose deposition has also been observed in response to infestation by phloem-feeding insects, such as the Russian wheat aphid *Diuraphis noxia*, whitefly nymphs, and brown planthopper. Some studies indicate that callose deposition in sieve element can interfere with feeding and provide resistance against these insects [31,54,55]. Additionally, research indicates that the deposition of *P. brassicae* eggs can induce callose accumulation in *Arabidopsis* and *B. nigra,* as well as upregulate a callose synthesis gene in *Arabidopsis* [56]. However, the precise role of callose in response to insect eggs remains unclear.

In this study, we observed significant callose deposition at the sites where whitefly egg pedicels were inserted into tobacco leaves (Figure 7a). Inhibiting callose deposition resulted in a significant increase in the egg-hatching rate (Figure 7a,b), underscoring the role of egg-induced callose deposition in plant resistance against eggs. Given that the whitefly eggs absorb sap from plant cells through their pedicels (Figure 1d), we speculate that callose deposition in those cells may hinder sap absorption. Further validation is needed to clarify the callose-mediated plant defense mechanisms in response to insect eggs.

Rivière et al. [57] demonstrated that silencing *PR-1a* in tobacco resulted in decreased callose deposition and increased β-1,3-glucanase activity. This report prompted us to investigate whether the resistance mechanism of *NtPR1* against eggs is related to callose. Our results indicate that overexpression of *NtPR1* in tobacco leads to increased callose deposition (Figure 7d, Figure 8). *Callose synthase* (*CalS*) genes are responsible for callose synthesis in plants, while β-1,3-glucanase catalyzes the degradation of callose [29]. We found that *NtPR1* overexpression did not affect the expression levels of *callose synthase* (*NtCalS*) genes, but significantly downregulated the expression of a *β-1,3-glucanase* (*NtBG*) gene (Figure S7). The reduction in *NtBG* expression may inhibit callose degradation, aligning with the observed increase in callose deposition following *NtPR1-L1* overexpression. This suggests that *NtPR1-L1* may promote egg-induced callose deposition by downregulating *NtBG* gene expression. Further exploration of the mechanisms underlying *PR1*-mediated callose accumulation is likely to provide valuable insights into plant defense strategies.

Additionally, since *NtPR1-L1* is activated by SA downstream *NtWRKY70a*, we further explored the roles of SA and *NtWRKY70a* in callose deposition. Previous studies have shown that SA can enhance disease resistance by triggering callose synthesis. In *Arabidopsis*, SA activates the expression of *CalS1* and *CalS8*, leading to callose biosynthesis that reduces viral cell-to-cell transport and restricts viral spread [33,58]. In this study, we found that SA application promotes callose deposition in tobacco (Figure 7c). Additionally, callose accumulation can also be induced by a microbial elicitor in *WRKY70*-overexpressing *Arabidopsis* plants [59,60], suggesting that WRKY70 positively regulates callose deposition. Consistent with this, our findings show that overexpression of *NtWRKY70a* also promotes callose deposition in tobacco (Figure S8), providing additional insights into the mechanisms of callose accumulation associated with plant stress adaptation. Moreover, another resistant protein, NtPR5-L2, is identified as a thaumatin-like protein. Osmotin, a component of this protein, has been reported to possess antifungal properties, altering fungal cell membrane permeability and causing cell membrane rupture [44,61]. Further research is warranted to investigate whether this protein can alter the membrane permeability of egg pedicel cells, potentially affecting whitefly eggs.

The eggs of another phloem-feeding insect, the brown planthopper, have been extensively studied and are shown to induce JA accumulation, producing substances that can kill the eggs in rice plants [40,62]. In contrast, whitefly eggs primarily induce SA without significantly increasing JA levels, and notable egg mortality in wide-type *Arabidopsis*, tobacco, tomato, and soybean plants has not been observed. The discrepancy may stem from the fact that planthopper oviposition causes severe damage to rice leaf sheaths, while whitefly egg laying results in minimal damage, which does not substantially weaken the plant, even with large numbers of eggs. It is also important to acknowledge the limitations of our study. Female whiteflies display behaviors where egg laying and feeding are difficult to differentiate. We aimed to minimize feeding effects in transcriptome design, but as males and females differ in their ability to feed on plant, it remains possible that the DEGs observed in the EF vs. F comparison reflect tobacco’s response to both female feeding and egg presence, rather than solely eggs.

## 4. Materials and Methods

### 4.1. Plants and Insects

Seeds of tobacco (*Nicotiana tabacum* cv. NC89, *Nicotiana benthamiana*) and cotton (*Gossypium hirsutum* L. cv. Zhemian 1793) were from our lab. Seeds of wild-type tobacco (*N. tabacum* var. Samsun NN) and transgenic NahG tobacco expressing the bacterial *salicylate hydroxylase* (*NahG*) gene were kindly provided by Prof. Han-Song Dong (Department of Plant Pathology, Nanjing Agricultural University, China). Seeds of transgenic *AOC*-RNAi tobacco with the JA biosynthetic gene *allene oxide cyclase* (*AOC*) silencing were kindly provided by Prof. Jian-Qiang Wu (Kunming Institute of Botany, Chinese Academy of Sciences, China).

*Bemisia tabaci* species MEAM1 (*mitochondrial cytochrome oxidase subunit I* GenBank accession no. GQ332577) was used in the study. Whiteflies were maintained on cotton plants in insect-proof cages. Growth chambers (25 ±  3°C, 60 ± 10% relative humidity, 16 h:8 h, light:dark) were used for all plant culturing and whitefly population rearing. All insect–plant experiments were also conducted in the growth chambers.

### 4.2. RNA-Seq

To investigate the plant response to whitefly eggs, we designed an experiment outlined in Figure 1e. Adult whiteflies newly emerged in 3–5 d were collected from colonies on cotton and divided into two groups based on sex. (a) Eggs + feeding (EF): 100 female whiteflies were released onto a single 3–4 true-leaf tobacco plant, and removed from the plant two days later to reduce the impact of feeding. After an additional 4 d, the leaves with eggs, usually the two lower leaves, were sampled, and the entire plant was checked to ensure no nymphs were present; (b) feeding (F): 100 males were released onto a 3–4 true-leaf tobacco plant, and removed two days later. After an additional 4 d, leaves from the same positions of each plant were sampled; (c) control (C): untreated tobacco plants. Almost 500 eggs were found on sampled leaves of each EF plant, while no eggs were present on the C and F. Each group contained three replicates, with each replicate consisting of samples from two tobacco plants. Total RNA of the samples for RNA-seq was extracted using Trizol reagent (Thermo Fisher Scientific, Waltham, MA) following the manufacturer’s instructions. The RNA libraries were sequenced on the Illumina Novaseq™ 6000 platform by LC Bio-Technology CO., Ltd. (Hangzhou, China). The RNA-seq raw data sets are available through the NCBI Sequence Read Archive (SRA) data library under project accession PRJNA1025342.

### 4.3. Differential Gene Expression Analysis and Enrichment Analysis

The fragments per kilobase of transcript per million mapped reads (FPKM) value was calculated by StringTie and Ballgown to obtain the estimated expression levels of all transcripts and perform expression abundance for mRNAs in the different samples [63]. Genes differential expression analysis between different groups was performed using DESeq2 software, and by edgeR between two samples [64,65]. For controlling the false discovery rate (FDR), Benjamini and Hochberg’s approach was used to adjust the *p*-values. Genes with an FDR-adjusted *p*-value < 0.05 and fold change ≥ 2 were considered as differentially expressed genes (DEGs). Gene Ontology (GO) enrichment analysis and the Kyoto Encyclopedia of Genes and Genomes database (KEGG) analysis of DEGs were then performed using the OmicStudio tools at https://www.omicstudio.cn/tool (accessed on 9 August 2024) [66].

### 4.4. Reverse Transcription and Quantitative PCR (RT-qPCR)

Total RNA for qRT-PCR was extracted from 100 mg tobacco leaves using *AG RNAex Pro* reagent (Accurate Biotechnology, Changsha, China). Complementary DNA (cDNA) was synthesized using an *Evo M-MLV* RT Kit (Accurate Biotechnology). qPCR was performed on the CFX96^TM^ Real-Time system (Bio-Rad, Hercules, CA, USA) with SYBR^®^ Premix Ex Taq II (TaKaRa, Dalian, China). All protocols were according to the manufacturer’s instructions. *NtGAPDH* was used as a housekeeping gene for transcript normalization. The relative gene expression level was determined using the 2^−ΔΔCt^ method. All primers are listed in Table S3.

### 4.5. Measurement of Salicylic Acid (SA), Jasmonic Acid (JA), and JA-Isoleucine (JA-Ile)

After the same processing and sampling method as for RNA-seq, leaves with eggs were collected and powdered in liquid nitrogen. Phytohormone extraction was performed using procedures as previously described [67], then analyzed with an Agilent 6460 triple quadrupole mass spectrometer (Agilent Technologies, Santa Clara, CA, USA) equipped with an electrospray ionization (ESI) source, operating in the negative or positive ion multiple-reaction monitoring (MRM) mode. Tobacco plants used were at the early 3–4 true-leaf stage. Each treatment had six replicates, and each replicate was sampled from one tobacco plant.

### 4.6. Exogenous Application of SA and Methyl Jasmonate (MeJA)

*Nicotiana tabacum* plants at the 3–4 true-leaf stage were subjected to treatments with either SA or MeJA in lanolin, at concentrations of 0.1 mM, 1 mM, and 10 mM, as described by Kessler and Baldwin [68]. Pure lanolin was used as the control. Each plant’s stem was treated with 1 mL of lanolin or a lanolin and phytohormone mixture once a day for 5 d. For RT-qPCR or callose staining, leaves at the same position were sampled.

### 4.7. Overexpression and Virus-Induced Gene Silencing (VIGS) of Genes in Tobacco

To overexpress *NtWRKY70* and *NtPR* genes in tobacco, the CDS of each gene was cloned into the plant expression vector pCAMBIA1305 using restriction enzymes (Thermo Fisher Scientific) and NovoRec^®^ plus One step PCR Cloning Kit (Novoprotein, Jiangsu, China). The recombinant plasmid was transformed into *Agrobacterium tumefaciens* strain EHA105 by electroporation. The transformed *A. tumefaciens* cultures were grown overnight at 28 °C, 200 rpm in LB liquid medium with 50 mg L^−1^ kanamycin and 50 mg L^−1^ rifampicin. The cultivated cells were harvested and resuspended in infiltration buffer (10 mM MES, 10 mM MgCl_2,_ and 200 μM acetosyringone, pH = 5.7) until OD_600_ reached 1.0. The suspension liquid was infiltrated into leaves of tobacco at the 4–5 true-leaf stage with a needleless syringe. After 48 h, the gene expression level was determined by RT-qPCR. Primers are listed in Table S3. The gene symbols of *NtPRs* and *NtWRKY70s* were listed in Table S4.

To silence *NtWRKY70* and *NtPRs* in tobacco, a VIGS assay was performed as previously described [69]. A ca. 300 bp gene fragment was amplified and cloned into 2mDNA1 vector, and the recombinant plasmid was transformed into EHA105. *Agrobacterium* cultures (OD_600_ = 1.0) carrying 2mDNA1-*NtWRKY70* or -*NtPR* plasmid was 1:1 (*v*/*v*) mixed with the helper virus tobacco curly shoot virus (TbCSV) and co-infiltrated into all leaves of tobacco plants at the 2–3 true-leaf stage. Control plants were co-infiltrated with *Agrobacterium* carrying the empty vector 2mDNA1 and TbCSV. After *ca*. 20 d, the gene silencing efficiency was determined by RT-qPCR. Primers are listed in Table S3.

### 4.8. Whitefly Bioassay

Newly emerged (3–5 days old) whiteflies (five males and five females) were collected and released into a clip cage fixed on the abaxial surface of the *N. tabacum* leaf. After 1 d infestation, adult whiteflies were removed carefully from each clip cage. Eggs were allowed to develop for another 6–7 d on the leaf, then the numbers of nymphs and unhatched eggs were counted under a stereoscope. For the gene-overexpressed assay, a whitefly bioassay was performed 24 h after infiltration. For the gene-silenced assay, a bioassay was performed ca. 25 d after infiltration.

### 4.9. Subcellular Localization

The CDS of *NtWRKY70*, with the stop codon removed, was cloned into the plant expression vector pCAMBIA1305 for fusion expression with GFP (green fluorescent protein). *Agrobacterium* cultures (OD_600_ = 1.0) carrying the pCAMBIA1305-*NtWRKY70a* plasmid were infiltrated into *N. benthamiana* leaves. pCAMBIA1305 empty vector was used as a control. After 48 h, GFP fluorescence was observed under a confocal microscope (Zeiss LSM710, Oberkochen, Germany).

### 4.10. Yeast One-Hybrid Assay

The promoter sequence ca. 2 kb of *NtPR1* and *NtPR5* were amplified from *N. tabacum* genomic DNA, and cloned into pAbAi vector, to generate bait constructs pAbAi-NtPR1pro and pAbAi-NtPR5pro. The bait plasmids were digested with BstB I (New England Biolabs, Beverly, MA), then integrated into Y1H Gold yeast strain. pAbAi-p53 was used as the control. The resulting yeast strains were selected on SD/-Ura medium with Aureobasidin A (AbA; TaKaRa), and the minimal inhibitory concentration of AbA for baits was determined. The CDS of *NtWRKY70* was inserted into the pGADT7 to generate the pGADT7-NtWRKY70a construct, then transformed into yeast strain Y1Hgold carrying bait plasmids respectively. pGADT7 was used as a control and also transformed into Y1Hgold with bait. The resulting yeast strains were selected on SD/-Leu, and transformed yeast cultures were grown overnight at 30°C, 200 rpm, in YPDA liquid medium. Cultivated cells were harvested and resuspended in sterile water until OD_600_ = 3.0, then cultured on SD/-Leu medium for 3 d, with the minimal inhibitory concentration of AbA for baits.

### 4.11. GUS-Staining Assay

The promoter sequences ca. 2  kb of *NtPR1* and *NtPR5* were cloned into the plant expression vector pBI121 as reporters *NtPR1pro::GUS* and *NtPR5pro::GUS*, respectively. pCAMBIA1305-NtWRKY70 plasmid was used as an effector, with the pCAMBIA1305 empty vector as a negative control. *Agrobacterium* cultures (OD_600_ = 1.0) carrying the reporter and effector constructs were 1:1 (*v*/*v*) co-infiltrated into *N. benthamiana* leaves. After 48 h, samples were detected by a GUS staining kit (Huayueyang, Beijing, China) according to the manufacturer’s protocols.

### 4.12. Dual-Luciferase Assay

The promoter sequences ca. 2  kb of *NtPR1-L1* and *NtPR5-L2* were cloned into the plant expression vector pGreenII0800-LUC as reporters *NtPR1pro::LUC* and *NtPR5pro::LUC*, respectively. *Renilla* luciferase (*REN*) driven by *35S* promoter in this vector was used as an internal control. Then, reporters were transferred into the *A. tumefaciens* strain EHA105. The effector was the same as that in the GUS staining assay. After co-infiltration of the reporter and effector into tobacco leaves for 2 d, the luciferase activities were measured using the Dual-Luciferase Reporter Assay Kit (Vazyme, Nanjing, China) by the FlexStation^®^3 Multi-Mode Microplate Reader (Molecular Devices, San Jose, CA). The relative luciferase activity was determined based on the ratio of LUC:REN bioluminescence.

### 4.13. Callose-Staining Assay

Callose staining was performed on the *N. tabacum* leaves following the method modified from Schenk and Schikora [70]. Diced leaves were incubated in fixative FAA (5% formalin, 5% acetic acid, 45% ethanol, and H_2_O) overnight, then incubated in 75% ethanol for destaining until transparent. After washing in sterile water three times, leaves were incubated in a staining solution (150 mM K_2_HPO4 and 0.01% aniline blue) for 1 h in darkness. Callose was observed with a Zeiss LSM710 confocal microscope using a UV filter. For detecting egg-induced callose deposition, adult whiteflies were collected and released to 2–3 true-leaf tobacco plants for 2 d, then leaves with eggs were sampled. For gene-overexpressed tobacco leaves, a staining assay occurred 2 d after infiltrating. To inhabit the callose synthesis, 5 mM 2-deoxy-D-glucose (2-DDG), a callose synthesis inhibitor, was injected into tobacco leaves, while sterile water served as a control [71,72,73]. Female adults were then introduced onto each treated leaf 1 day after, and leaf samples were collected after an additional 2 d.

## Data Availability

The original contributions presented in the study are included in the article/Supplementary Material, further inquiries can be directed to the corresponding author.

## Supplementary Materials

The following supporting information can be downloaded at: https://www.mdpi.com/article/10.3390/ijms252212076/s1.
