# Supplementary material for: The SA-WRKY70-PR-Callose Axis Mediates Plant Defense Against Whitefly Eggs
Source: Int J Mol Sci. 2024 Nov 10;25(22):12076. doi: 10.3390/ijms252212076 (PMC11593482; doi:10.3390/ijms252212076)
Supplement: Supplementary file 1 [file ijms-25-12076-s001.zip › Document S1-v2.pdf]

Supplementary Materials：

The SA-WRKY70-PR-callose axis mediates plant defense against whitefly eggs

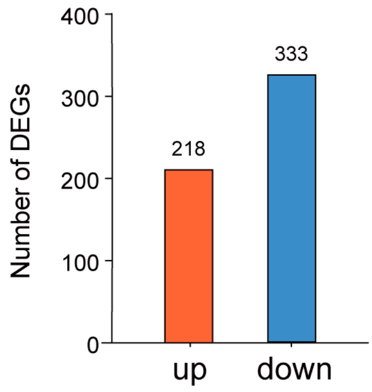

Figure S1. Tobacco transcriptome responses to feeding treatment.

Histogram showing up- and down-regulated number of DEGs between feeding samples (F) vs. control samples (Control).

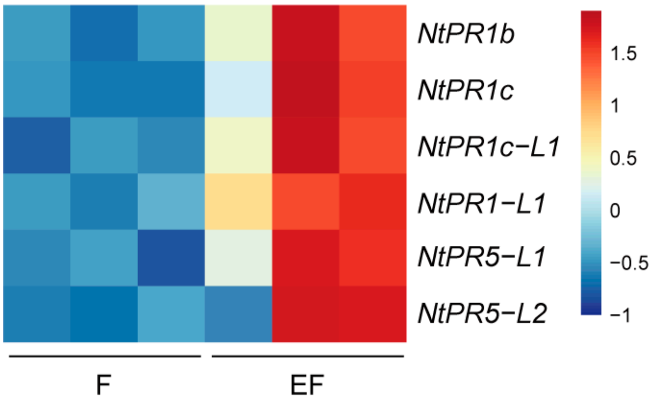

Figure S2. Expression pattern of *NtPRs* in transcriptome.

Heatmap showing the expression pattern of 6 *NtPR* genes in transcriptome between feeding samples (F) and egg + feeding samples (EF). Each treatment has three replicates. The red to blue colors represent high to low expression levels based on the Log10-transformed FPKM values.

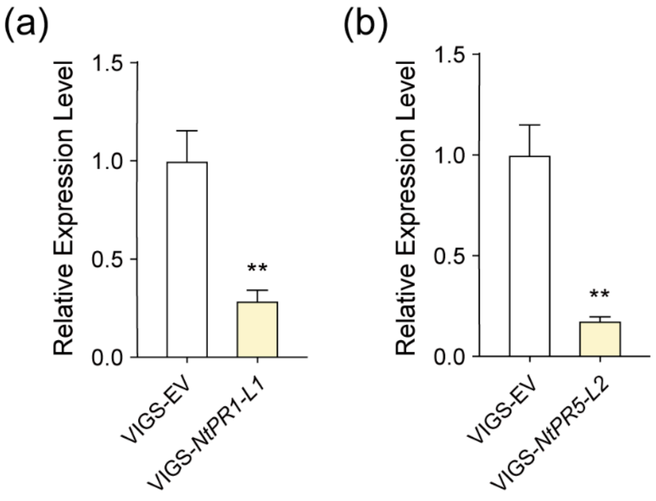

Figure S3. The efficiency of *NtPRs* silencing by VIGS.

The relative transcript levels of *NtPRs* in control and *NtPRs*-silenced tobacco. Values are mean ± SEM, n = 20. **, P < 0.01 (Student's t-test).

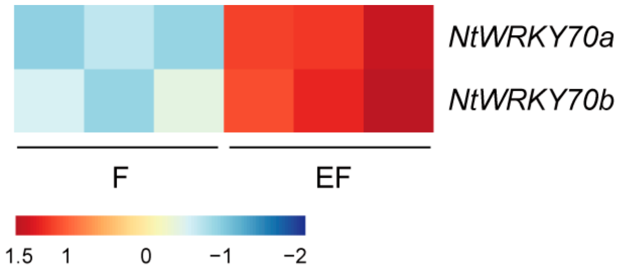

Figure S4. Expression pattern of *NtWRKY70s* in transcriptome.

Heatmap shows the expression pattern of two *NtWRKY70-like* genes in transcriptome between feeding samples (F) and egg + feeding samples (EF). Each treatment had three replications. The red to blue colors represent high to low expression levels based on the Log10-transformed FPKM values.

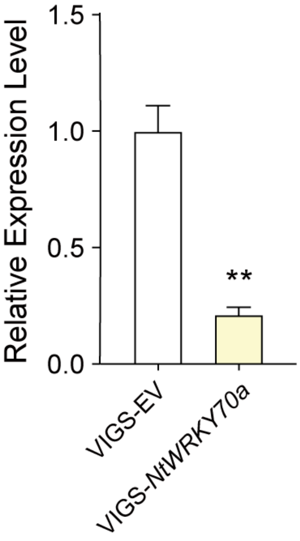

Figure S5. The efficiency of *NtWRKY70a* silencing by VIGS.

The relative transcript levels of *NtWRKY70a* in control and VIGS-*NtWRKY70a* tobacco. Values are mean ± SEM, $n = 20$. **, $P < 0.01$ (Student's *t*-test).

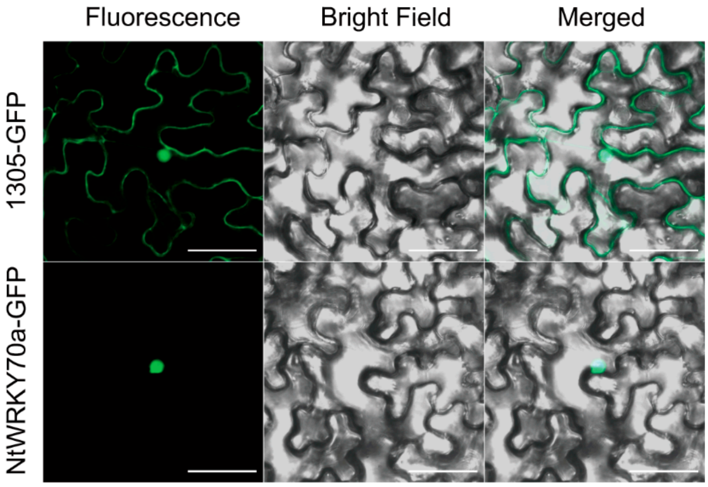

Figure S6. Subcellular localization of NtWRKY70a in *Nicotiana benthamiana* leaves. Bars, 50 μm.

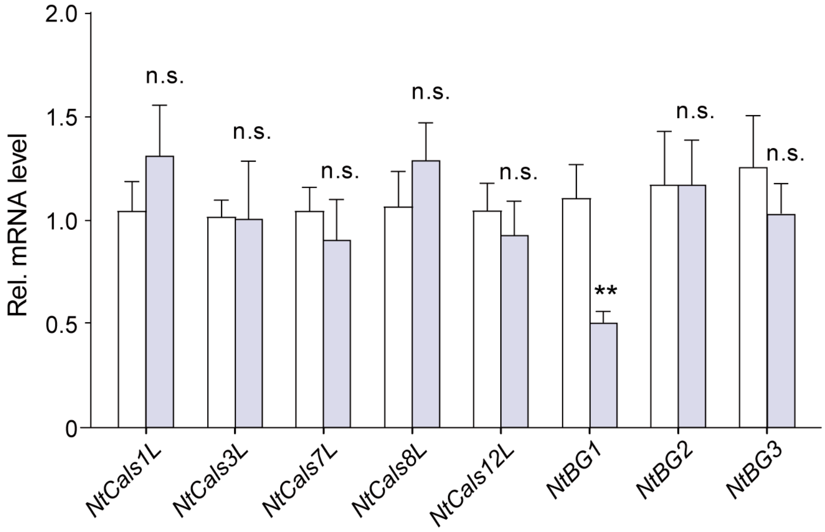

Figure S7. The relative expression level of *NtCalS and NtBG* genes in OE-*NtPR1-L1* plants and control.

CalS, callose synthase gene. BG, β-1,3-glucanase. OE, overexpression. Values are mean ± SEM, n = 6–8. n.s., not significant; **, $P < 0.01$ (one-way ANOVA, LSD test).

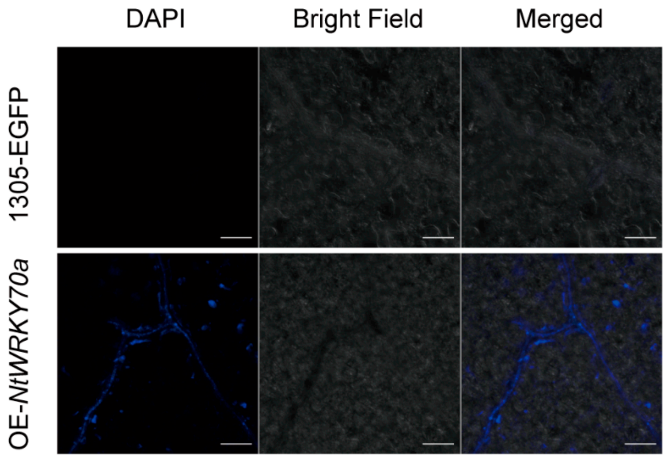

Figure S8. Callose staining assay of OE-*NtWRKY70a* tobacco and control. Bars, 50 μm.

Table S1. DEGs between eggs + feeding (EF) and feeding (F).

Too long to be displayed here, please check it in another document in excel type.

Table S2. Expression level of SA- and JA-related genes in egg + feeding (EF) compared with feeding (F).

Gene symbol	Function	Fold Change	FDR	Regulation
**SA-related Genes**				
LOC107762275	phytoalexin deficient 4 (PAD4)	3.8	0.00520	up
LOC107768730	phytoalexin deficient 4 (PAD4)	2.9	0.01318	up
LOC107794034	isochorismate synthase 1 (ICS1)	0.9	0.18498	-
LOC107775128	isochorismate synthase 1 (ICS1)	0.8	0.28078	-
LOC107811391	enhanced disease susceptibility 1L (EDSL)	4.8	0.00000	up
LOC107782626	enhanced disease susceptibility 1L (EDSL)	2.9	0.00112	up
LOC107800256	phenylalanine ammonia-lyase (PAL)	5.9	0.00000	up
LOC107800257	phenylalanine ammonia-lyase (PAL)	7.9	0.00002	up
LOC107821864	nonexpresser of PR genes (NPR1)	2.2	0.02654	up
LOC107779651	myb domain protein 48 (MYB48)	5.0	0.00000	up
LOC107824441	mitogen-activated protein kinase (MAPK)	2.7	0.00293	up
LOC107826288	mitogen-activated protein kinase (MAPK)	2.9	0.00131	up
LOC107800971	glutaredoxin (GRX)	5.6	0.00055	up
**JA-related Genes**				
LOC107785525	phospholipase (PLA)	1.3	0.89969	-
LOC107820064	lipoxygenase (LOX)	1.6	0.32978	up
LOC107783985	allene oxide synthase (AOS)	1.3	0.90076	-
LOC107832778	allene oxide synthase (AOS)	1.6	0.52513	-

LOC107768393	allene oxide cyclase (AOC)	1.2	0.95587	-
LOC107767576	allene oxide cyclase (AOC)	1.0	0.40377	-
LOC107782790	12-oxophytodienoate reductase 3 (OPR3)	1.3	0.81505	-
LOC107773112	12-oxophytodienoate reductase 3 (OPR3)	1.2	0.95251	-
LOC107807744	3-ketoacyl-CoA thiolase (KAT)	1.4	0.68348	-
LOC107767049	transcription factor MYC2 (MYC2)	1.4	0.55357	-
LOC107820916	transcription factor MYC2 (MYC2)	1.1	0.76807	-
LOC107792348	jasmonate-zim-domain protein (JAZ)	1.8	0.60889	-

Table S3. Primers used in this study.

Primer		Sequences (5'-3')	Purpose
**qPR1b**	**F**	CGAGATGTGGGTCGATGAGA	qRT-PCR for *NtPR1b*
	**R**	GTTACGCCAAACCACCTGAG	
**qPR1c**	**F**	CACAGCTCGTGCAGATGTAG	qRT-PCR for *NtPR1c*
	**R**	GCAGCCAATTGGGAAGCATA	
**qPR1c-1**	**F**	GCTAAGGCCGTTGAGATGTG	qRT-PCR for *NtPR1c-L1*
	**R**	GAGTTACGCCAAACCACCTG	
**qPR1-L1**	**F**	TCTTGGTTGTGCTAGGGTCA	qRT-PCR for *NtPR1-L1*
	**R**	CTGCAAGATCACCGTAGGGA	
**qPR5-L1**	**F**	TGAACCCAGGAACAGTCCAG	qRT-PCR for *NtPR5-L1*
	**R**	TCCCGTTACAGTCTCCAGTC	
**qPR5-L2**	**F**	TGAACCCAGGAACAGTCCAG	qRT-PCR for *NtPR5-L2*
	**R**	AGCATCCCGTTACAGTCTCC	
**PR1b OE**	**F**	AAGTCCGGAGCTAGCTCTAGAATGGGATTTTTCTCTTTTCACA	Overexpression of *NtPR1b*
	**R**	GCCCTTGCTCACCATGGATCCGTATGGACTTTGGCCTATGACA	
**PR1c OE**	**F**	AAGTCCGGAGCTAGCTCTAGAATGGAATTTGTTCTCTTTTCACAAATG	Overexpression of *NtPR1c*
	**R**	GCCCTTGCTCACCATGGATCCGTATGGGCTTTTGCCTATAACATTAC	
**PR1c-L1 OE**	**F**	AAGTCCGGAGCTAGCTCTAGAATGGGATTTGTTCTCTTTTCCCAAAT	Overexpression of *NtPR1c-L1*
	**R**	GCCCTTGCTCACCATGGATCCAGCCGAAGTCCGTACATATATAACG	

**PR1-L2 OE**	**F**	AAGTCCGGAGCTAGCTCTAGAATGGGATACTCCACAACATTAGT	Overexpression of *NtPR1-L1*
	**R**	GCCCTTGCTCACCATGGATCCTACATCAGTTGGAAGTTCCAACT	
**PR5-L2 OE**	**F**	AAGTCCGGAGCTAGCTCTAGAATGAACTTCCTCAAAAGCTTCCC	Overexpression of *NtPR5-L2*
	**R**	GCCCTTGCTCACCATGGATCCAGGGCAGAAGACAACCCTGT	
**qPR1 VIGS**	**F**	TAACGTATTTATACTCGGATCCTATATTATTTCCCTCATCTCAAGCTCA	VIGS of *NtPR1-L1*
	**R**	GTATTTGGACTAAATACTCTAGACGGCTAGGTTTTCGCCGTAA	
**qPR5 VIGS**	**F**	TAACGTATTTATACTCGGATCCATGAACTTCCTCAAAAGCTTCCC	VIGS of *NtPR5-L2*
	**R**	GTATTTGGACTAAATACTCTAGACCCCAAATGCGAGCCTGG	
**qPR1 se**	**F**	CGCTGGTGCCGTAAAAATGT	qRT-PCR for *NtPR1-L1*silencing
	**R**	TTGCTCTGCAAGATCACCGT	efficiency detection
**qPR5 se**	**F**	CCCATGGAATTCAGCCCAAC	qRT-PCR for *NtPR5-L2* silencing
	**R**	GCTGGGCATTGCTCGTTAAT	efficiency detection
**qWRKY70a**	**F**	GGAGGGAAAGACCGTAGAGG	qRT-PCR for *NtWRKY70a*
	**R**	GTGGGTGCACCTAAAGTAGC	
**qWRKY70b**	**F**	ACGGTCAGAAGCACATTCTC	qRT-PCR for *NtWRKY70b*
	**R**	TGACTCGAAATAGTGGCGGAT	
**WRKY70a OE**	**F**	AAGTCCGGAGCTAGCTCTAGAATGAAGAAGCCATTAGTTCATGAAAAC	Overexpression of *NtWRKY70a*
	**R**	GCCCTTGCTCACCATGGATCCAGAAAACTCTATGGCCTCATCAAAGT	
**WRKY70b OE**	**F**	AAGTCCGGAGCTAGCTCTAGAATGGAGTCTGAAAATTACACACAGT	Overexpression of *NtWRKY70b*
	**R**	GCCCTTGCTCACCATGGATCCAAATTGAAAAGATAAATCAGCA	

**qWRKY70a VIGS**	**F**	TAACGTATTTATACTCGGATCCGCTTGCAACTCACTAAAGATTACAAAGCA	VIGS of *NtWRKY70*a
	**R**	GTATTTGGACTAAATACTCTAGAACATCATTAGAAACATCATCCCTTTGTACC	
**qWRKY70a se**	**F**	AATCAAAGAACTGGTTGACGGA	qRT-PCR for *NtWRKY70* silencing
	**R**	GATCTCCAAATTTTGAGAAGAAGCTCA	efficiency detection
**pAbAi-PR1pro**	**F**	AAATGATGAATTGAAAAGCTTTGAGGGTTTTAGGGTACACGG	Construction of pAbAi-NtPR1pro bait
	**R**	GTCGACAGATCCCCGGGTACCAAGGAATAGAGTGGGTGGGT	vector
**pAbAi-PR5pro**	**F**	AAATGATGAATTGAAAAGCTTCTGCGGTGAGGAGAGTAGCA	Construction of pAbAi-NtPR5pro bait
	**R**	GTCGACAGATCCCCGGGTACCGACACTATCGATGGGTTTAAATAGCT	vector
**pGADT7-WRKY70**	**F**	GCCATGGAGGCCAGTGAATTCAAGAAGCCATTAGTTCATGAAAACCC	Expression of *NtWRKY70a* in Y1H Gold
	**R**	CAGCTCGAGCTCGATGGATCCAGAAAACTCTATGGCCTCATCAAAG	
**PBI121-PR1pro**	**F**	GACCATGATTACGCCAAGCTTTGAGGGTTTTAGGGTACACGG	Construction of PBI121-PR1pro reporter
	**R**	GGACTGACCACCCGGGGATCCAAGGAATAGAGTGGGTGGGT	vector
**PBI121-PR5pro**	**F**	GACCATGATTACGCCAAGCTTCTGCGGTGAGGAGAGTAGCA	Construction of PBI121-PR5pro reporter
	**R**	GGACTGACCACCCGGGGATCCGACACTATCGATGGGTTTAAATAGCT	vector
**Green-PR1pro**	**F**	CTATAGGGCGAATTGGGTACCTGAGGGTTTTAGGGTACACGG	Construction of pGreenII0800-PR1pro
	**R**	TGTTTTTGGCGTCTTCCATGGAAGGAATAGAGTGGGTGGGT	reporter vector
**Green-PR5pro**	**F**	CTATAGGGCGAATTGGGTACCCTGCGGTGAGGAGAGTAGCA	Construction of pGreenII0800-PR5pro
	**R**	TGTTTTTGGCGTCTTCCATGGGACACTATCGATGGGTTTAAATAGCT	reporter vector
**qCalS1**	**F**	GGAGCTTTGACAGAATGTGGA	qRT-PCR for *NtCals1L*
	**R**	GAAACTTGCCCGAGCTTCAA	

**qCalS3**	**F**	GGGAGGAGGAGGCTTTCTTT	qRT-PCR for *NtCals3L*
	**R**	GCCAACCTAACCTGAAGCAG	
**qCalS7**	**F**	ACTTCCTGTCAGTGGAGGTG	qRT-PCR for *NtCals7L*
	**R**	TGTCCCACTCTGGTTTCTCC	
**qCalS8**	**F**	ACTTGGCATGAGAGAGCACA	qRT-PCR for *NtCals8L*
	**R**	ATGGAACCTGACCCTGAGTG	
**qCalS12**	**F**	ACTTTCGACTTGTGCAGCTC	qRT-PCR for *NtCals12L*
	**R**	GCCAACAGGCTGATGAAGAG	
**qBG1**	**F**	AGGGATTGTTGTGTCCGAGA	qRT-PCR for *NtBG1*
	**R**	TAGGTCCAGGCTTTCTTGGG	
**qBG2**	**F**	CACGTTTCCAACACTGCTGA	qRT-PCR for *NtBG2*
	**R**	TAGTTGCTGCAGAGCTTCCT	
**qBG3**	**F**	CACCTACCCACCCAAAGCTA	qRT-PCR for *NtBG3*
	**R**	CCTCGTTGTGTGAACAATGC	
**qGAPDH**	**F**	GCAGTGAACGACCCATTTATCTC	qRT-PCR for *NtGADPH*
	**R**	AACCTTCTTGGCACCACCCT	

Table S4. Gene symbols and names of *NtPRs* and *NtWRKY70s*.

Gene symbol	Gene name in this paper
LOC107807832	*NtPR1b*
LOC107763263	*NtPR1c*
LOC107808770	*NtPR1c-L1*
LOC107798618	*NtPR1-L1*
LOC107776518	*NtPR5-L1*
LOC107769943	*NtPR5-L2*
LOC107794474	*NtWRKY70a*
LOC107812348	*NtWRKY70b*
